# Hypogonadism and liver fibrosis in HIV-infected patients

**DOI:** 10.1007/s40618-021-01512-9

**Published:** 2021-01-29

**Authors:** E. Quiros-Roldan, T. Porcelli, L. C. Pezzaioli, M. Degli Antoni, S. Paghera, M. Properzi, E. Focà, C. Carriero, F. Castelli, A. Ferlin

**Affiliations:** 1 Department of Infectious and Tropical Diseases, University of Brescia and ASST Spedali Civili Hospital, Brescia, Italy; 2 Endocrinology, Montichiari Hospital, ASST Spedali Civili Brescia, Montichiari, Brescia, Italy; 3 Unit of Endocrinology and Metabolism, Department of Clinical and Experimental Sciences, University of Brescia and ASST Spedali Civili Brescia, Viale Europa 11, 25123 Brescia, Italy; 4 Diagnostic Department, ASST Centro di Ricerca Emato-oncologica AIL (CREA), Spedali Civili di Brescia, Brescia, Italy

**Keywords:** Hypogonadism, HIV, Liver fibrosis, Testosterone, LH, SHBG

## Abstract

**Purpose:**

Hypogonadism is frequent in HIV-infected men and might impact on metabolic and sexual health. Low testosterone results from either primary testicular damage, secondary hypothalamic-pituitary dysfunction, or from liver-derived sex-hormone-binding-globulin (SHBG) elevation, with consequent reduction of free testosterone. The relationship between liver fibrosis and hypogonadism in HIV-infected men is unknown. Aim of our study was to determine the prevalence and type of hypogonadism in a cohort of HIV-infected men and its relationship with liver fibrosis.

**Methods:**

We performed a cross-sectional retrospective study including 107 HIV-infected men (median age 54 years) with hypogonadal symptoms. Based on total testosterone (TT), calculated free testosterone, and luteinizing hormone, five categories were identified: eugonadism, primary, secondary, normogonadotropic and compensated hypogonadism. Estimates of liver fibrosis were performed by aspartate aminotransferase (AST)-to-platelet ratio index (APRI) and Fibrosis-4 (FIB-4) scores.

**Results:**

Hypogonadism was found in 32/107 patients (30.8%), with normogonadotropic (10/107, 9.3%) and compensated (17/107, 15.8%) being the most frequent forms. Patients with secondary/normogonadotropic hypogonadism had higher body mass index (BMI) (*p* < 0001). Patients with compensated hypogonadism had longer HIV infection duration (*p* = 0.031), higher APRI (*p* = 0.035) and FIB-4 scores (*p* = 0.008), and higher HCV co-infection. Univariate analysis showed a direct significant correlation between APRI and TT (*p* = 0.006) and SHBG (*p* = 0.002), and between FIB-4 and SHBG (*p* = 0.045). Multivariate analysis showed that SHBG was independently associated with both liver fibrosis scores.

**Conclusion:**

Overt and compensated hypogonadism are frequently observed among HIV-infected men. Whereas obesity is related to secondary hypogonadism, high SHBG levels, related to liver fibrosis degree and HCV co-infection, are responsible for compensated forms.

## Introduction

Hypogonadism is a clinical syndrome characterized by low testosterone (T) plasma levels with symptoms and signs of low androgen action, caused by alteration of the hypothalamus–pituitary-testis axis (HPT) at one or more stages [1, 2]. Sexual symptoms, such as decreased libido and erectile dysfunction, are considered the most specific manifestations, but many other conditions and co-morbidities are associated with hypogonadism, for example osteoporosis, increased fat/lean mass, cardiovascular diseases, sarcopenia, asthenia, and depression [3]. Hypogonadism is a relatively common finding in men with HIV, although its prevalence is at present lower comparing to the pre-antiretroviral therapy (ART) era [4, 5]. Current estimates on the prevalence of hypogonadism in HIV-infected men generally range from 13 to 40% according to different study settings, mainly depending on the criteria and T-levels cut-offs used for its diagnosis [6, 7]. Importantly, hypogonadism in HIV-infected men might result from either testicular disorder (primary hypogonadism) or hypothalamic-pituitary dysfunction (secondary hypogonadism); however, only few studies differentiated between these two forms by testing luteinizing hormone (LH) levels, which are elevated in the former case and low/normal in the latter. Furthermore, compensated hypogonadism, which represents another frequent finding in both, general population and HIV-infected men complaining of sexual dysfunction, can be diagnosed by LH determination, since it is characterized by normal T levels and high LH [8–10]. Hypogonadism can also be linked to sex-hormone binding protein (SHBG) alteration. In fact, androgen actions are mediated by free T (FT), which represents only 1–3% of total T, being the large part bound to albumin and SHBG, both produced by the liver. Therefore, in conditions in which SHBG is high (as liver diseases or HIV infection), determination of FT is necessary because total T (TT) might be apparently normal [11]. Moreover, methods that directly measure FT are inaccurate; the best approach is represented by its calculation (calculated FT, cFT) after determination of SHBG and albumin using formulae, as the Vermeulen one (http://www.issam.ch/freetesto.htmwww.issam.ch/freetesto.htm).

However, liver function, SHBG, LH and FT levels have been rarely evaluated together in HIV-infected men. Interestingly, the liver and the reproductive system are bidirectionally linked. Sex steroid signaling influences hepatic metabolism and contributes to the pathogenesis of functional and structural disorders of the liver [12]. In turn, liver function affects the reproductive axis modulating not only SHBG levels, but also sex steroid metabolism [13]. In the general population T is reduced in up to 90% of men with cirrhosis [12] and in male patients with HCV-related chronic liver diseases [14]. In addition, the relationship between sex hormones and SHBG levels and Non Alcoholic Steato Hepatitis (NASH), Non Alcoholic Fatty Liver Disease (NAFLD) and liver fibrosis has been reported in some studies [15–21], although with non-conclusive data.

In people living with HIV many overlapping factors contribute to the development of liver injury and progressive hepatic fibrosis, as HCV, HBV or HDV co-infections, alcohol or drugs abuse, chronic ART and fatty liver disease. Therefore, an accurate study regarding hypogonadism and liver fibrosis in men with HIV-infection is warranted.

The aim of this study was the determination of the prevalence and type of hypogonadism based on cFT, TT, SHBG and LH values in a cohort of middle-aged HIV-infected males with symptomatic hypogonadism and its relationship with liver fibrosis measured by Fibrosis-4 (FIB-4) and aspartate aminotransferase (AST)-to-platelet ratio index (APRI) scores.

## Methods

### Setting and study population

A cross-sectional observational retrospective study was performed. One-hundred and seven HIV-infected male patients under ART followed at our Clinic with hypogonadal symptoms (erectile dysfunction, reduced libido and morning spontaneous erections) were selected from a database of patients who underwent endocrinological evaluation from January 2012 to December 2019. Inclusion criteria were: age > 18 years, HIV infection under ART in stable clinical condition, sexual symptoms and availability of complete data for TT, cFT (calculated with the Vermeulen formula), SHBG, LH, AST, alanine aminotransferase (ALT), gamma-glutamyl transferase (GGT), platelets (PLT), and HCV data (Ab and RNA) from the same reference laboratory. TT and LH were determined using chemiluminescence microparticle immunoassay (CMIA), SHBG using chemiluminescence immunoassay (CLIA). Exclusion criteria were previous or current use of drugs affecting HPT axis, acute illnesses, known pituitary and testicular diseases, cancer, diabetes mellitus, chronic renal failure, laboratory data not coming from the reference laboratory of our Hospital. Demographic and clinical characteristic were recorded from clinical charts.

According to guidelines [22], patients were grouped by their HPT status in five categories: (a) eugonadism, defined as normal TT (> 3.46 ng/ml), cFT (> 65 pg/ml), and LH (> 1.5 < 9.4 IU/l); (b) compensated hypogonadism, defined as normal TT (> 3.46 ng/ml) and cFT (> 65 pg/ml) and elevated LH (> 9.4 IU/l); (c) secondary hypogonadism, defined as low TT (≤ 3.46 ng/ml) or cFT (≤ 65 pg/ml) and low LH (≤ 1.5 IU/l); (d) primary hypogonadism, defined as low TT (≤ 3.46 ng/ml) or cFT (≤ 65 pg/ml) and elevated LH (> 9.4 IU/l); (e) normogonadotropic hypogonadism, defined as low TT (≤ 3.46 ng/ml) or cFT (≤ 65 pg/ml) and normal LH (> 1.5 < 9.4 IU/l).

Estimates of liver fibrosis were performed by the APRI score [23], calculated as [AST (U/l)/upper normal × 100/platelet count (10^9^/l)], available at https://www.hepatitisc.uw.edu/page/clinical-calculators/apri, and the FIB-4 score [24], calculated as [Age (years) × AST (U/l)/[PLT (10^9^/l) × ALT1/2 (U/l)], available at https://www.hepatitisc.uw.edu/page/clinical-calculators/fib-4.

### Statistical analysis

GraphPad Prism version 5.1 (GraphPad Software, San Diego, CA) was used for statistical analysis. Comparison among medians of the quantitative variables were performed by non-parametric Kruskal–Wallis *H* test, since the variables were not normally distributed (D’Agostino and Pearson omnibus normality test was used). When the interaction was significant, post hoc Dunn’s test corrected *p* values were calculated. The Spearman’s correlation was calculated to verify the possible association with TT and cFT of the variables used. The association between liver fibrosis scores (FIB-4 and APRI) and age, BMI, HIV duration, HCV ab, and hormonal data was analysed with univariate and multivariate regression models. *p* values ≤ 0.05 were considered significant.

### Ethics

This study was conducted according to the Declaration of Helsinki and to principles of Good Clinical Practice (GCP). As this study had a retrospective design and was based on routinely collected data, patients’ informed consent was not required according to the Italian law (Italian Guidelines for classification and conduction of observational studies, established by the Italian Drug Agency, “Agenzia Italiana del Farmaco—AIFA” on March 20, 2008). Moreover, the study protocol was approved by the local Ethic Committee of Brescia Province (Comitato Etico di Brescia—August 2020 NP 3898). Data were analyzed anonymously, and each subject was identified using an alphanumerical code.

All authors had access to the study data and reviewed and approved the final manuscript.

## Results

### Patients’ characteristics

One hundred and seven patients were included in the study. Table 1 summarize their characteristics. The median age was 54 years (IQR 48–58) and median body mass index (BMI) 25 (IQR 23–27). 40% were smokers and 41% were HCV Ab positive (of which 61% with a detectable plasmatic HCVRNA). With respect to liver function, the proportion of patients with AST, ALT and GGT above upper normal limits was 14/107 patients (13.1%), 31/107 (28.9%) and 27/107 (25.2%), respectively. Median APRI and FIB-4 score were 0.3 (IQR 0.2–0.4) and 1.1 (IQR (0.8–1.5), respectively. The proportion of patients with severe fibrosis defined as APRI score > 1 or FIB-4 score > 2.67 were 16.6% (18/107) and 14% (15/107), respectively.

**Table 1:** Characteristics of the study population

	Normal range	Overall	Secondary—normogonadotropic hypogonadism	Compensated hypogonadism	Primary hypogonadism	Eugonadism	*p* value
		(# = 107)	(# = 12)	(# = 17)	(# = 3)	(# = 75)	
Age (years); median (IQR)		54 (48–58)	51 (46–57)	55 (49–58)	61 (54–64)	54 (48–58)	0.3291
BMI [median (IQR)]		25 (23–27)	26 (25–31)	24 (23–27)	25 (24–30)	25 (23–27)	0.2999
> 30; # (%)		14 (13)	8 (67)	1 (6)	1 (33)	4 (5)	< 0.0001
Smoker; # (%)		43 (40)	3 (25)	9 (53)	1 (33)	30 (40)	0.5008
Diabetic; # (%)		14 (13)	2 (17)	4 (24)	1 (33)	7 (9)	0.2867
HCV Ab positive; # (%)		44 (41)	5 (42)	11 (65)	3 (100)	25 (33)	0.0179
HCV RNA positive^a^; # (%)		27 (61)	2 (17)	10 (91)	1 (33)	14 (56)	0.0975
AST; median (IQR)	18–39	21 (16–30)	20.5 (14–27)	29.0 (20–45)	16.0 (16–31)	21.0 (16–29)	0.2215
ALT; median (IQR)	15–47	34 (26–50)	38 (31.5–54)	48 (30.5–63.5)	26 (18–51)	32 (25–45)	0.1063
GGT; median (IQR)	10–71	44 (27–86)	57 (21.8–142)	48 (31–89)	39 (26–48)	44 (25.5–84)	0.7317
PLT; median (IQR)	130–400	190 (161–233)	230 (186.5–275.5)	200 (142–246.5)	162 (145–175)	188 (156–230)	0.1140
TT; median (IQR)	3–9	6.5 (5.2–8.5)	4.4 (2.8–4.6)	7 (6–8.6)	3.3 (2.5–4.2)	6.7 (5.4–8.6)	0.0001
cFT; median (IQR)	65–260	101 (70.2–122.3)	54.8 (21.8–61.7)	108 (68.4–146)	41.4 (32.6–55)	108.5 (87.6–128)	< 0.0001
SHBG; median (IQR)	10–70	60 (41–87)	56 (36.5–99)	90 (52.9–107.5)	43 (41.6–117)	59.5 (38.8–69.8)	0.5906
LH; median (IQR)	1.5–9.0	5.6 (3.5–8.6)	3.9 (2.3–7.5)	15.7 (10.7–26.8)	23.6 (11.2–30.5)	4.6 (3–6.9)	< 0.0001
FSH; median (IQR)	1.5–8.0	5.9 (4.2–9.5)	5.2 (3.1-.15.7)	10 (6.2–16.4)	33.8 (15.9–51.8)	5.5 (4–7.9)	0.0021
APRI							
Mean (SD)		0.5 (0.9)	0.75 (1.7)	1.0 (1.5)	0.3 (0.2)	0.4 (0.3)	0.0352
Median (IQR)		0.3 (0.2–0.4)	0.2 (0.13–0.38)	0.40 (0.2–1.1)	0.20 (0.2–0.5)	0.3 (0.2–0.4)	0.3412
FIB-4							
Mean (SD)		1.5 (1.6)	1.5 (2.4)	2.6 (2.9)	1.4 (0.2)	1.2 (0.7)	0.0084
Median (IQR)		1.1 (0.8–1.5)	0.73 (0.53–1.3)	1.0 (0.69–5.17)	1.42 (1.15–1.62)	1.05 (0.81–1.37)	0.1518
Years with HIV; median (IQR)		18 (12–25)	15 (13–17)	30 (19–33)	22 (11–24)	17 (11–24)	0.0137
Years of ART; median (IQR)		15 (10–23)	14 (10–17)	23 (13–24)	22 (11–22)	13 (8–23)	0.0830
CD4/µl; median (IQR)		636 (456–793)	559 (506–682)	636 (537–799)	376 (256–1272)	660 (350–829)	0.7708
CD4%; median (IQR)		30.5 (23.9–37.6)	32.1 (26.8–42.6)	30.4 (26.5–38.3)	16.5 (14.7–36.6)	31.3 (23.5–35.6)	0.3402
CD4 nadir/µl; median (IQR)		125 (49–281)	184 (24–344)	125 (46–261)	63 (21–290)	126 (54–294)	0.8885
CD8/µl; median (IQR)		774 (524–1099)	697 (432–922)	798 (599–1062)	1294 (941–1533)	774 (521–1116)	0.1584
CD8%; median (IQR)		39.6 (31.9–46.7)	36.9 (33.9–44.7)	40.2 (23.7–46.5)	59.7 (37.2–60.9)	39.6 (31.3–49.5)	0.2845
CD4/CD8 ratio; median (IQR)		0.80 (0.52–1.14)	0.82 (0.63–1.21)	0.8 (0.6–0.95)	0.27 (0.25–0.98)	0.79 (0.5–1.26)	0.4330
AIDS; # (%)		31 (29)	4 (33)	5 (29)	2 (66)	20 (27)	0.4978

*BMI* body mass index (kg/m^2^), *NA* not available, *AST* aspartate transaminase (U/l), *ALT* alanine transaminase (U/l), *GGT* gamma-glutamyl transferase (U/l), *PLT* thrombocyte (× 10^3^/µl), *TT* total testosterone (ng/ml), *cFT* free testosterone (pg/ml), *SHBG* sex hormone binding globulin (nmol/l), *LH* luteinizing hormone (IU/l), *FSH* follicle-stimulating hormone (IU/l), *APRI* the aspartate aminotransferase (AST)-to-platelet ratio index, *FIB-4* fibrosis-4 index, *ART* antiretroviral therapy

*p* value was done by Kruskal–Wallis *H* test for continuous variables and Chi-square test for categorical variables. For APRI and FIB-4 mean values, *p* value was done by one-way analysis of variance and Bonferroni's multiple comparison test

^a^HCV RNA positive patients are calculated from the number of HCV Ab positive patients

### Hypogonadism and liver fibrosis

The prevalence of overt hypogonadism (defined as patients with low level of TT and/or cFT and including primary, secondary and normogonadotropic hypogonadism) was 14% (15/107). Normogonadotropic hypogonadism was the most frequent type (10/107, 9.3%). An additional 15.8% (17/107) had compensated hypogonadism (Table 1). Analyses were performed grouping together secondary and normogonadotropic hypogonadism (Table 1).

Patients with secondary/normogonadotropic hypogonadism were significantly more obese (BMI > 30) with respect to patients with eugonadism and primary and compensated hypogonadism (*p* < 0001). Patients with compensated hypogonadism had longer duration of HIV positivity (*p* = 0.031) and higher APRI (*p* = 0.035) and FIB-4 indexes (*p* = 0.008) compared to patients with eugonadism and primary and secondary/normogonadotropic hypogonadism (Table 1). Moreover, although not statistically different to the other groups, they tended to have more frequently a detectable HCVRNA and showed higher SHBG and transaminases. The low number of patients with primary hypogonadism (*n* = 3) did not allow to perform analyses on this group.

Table 2 shows reproductive hormonal levels according to liver fibrosis scores upper or under the mean. SHBG values were higher in patients with APRI score upper the mean (102 nmol/l vs 54 nmol/l in patients with APRI score below the mean; *p* = 0.004) and a trend for difference also observed when compared according to FIB-4 score (66 nmol/l vs 56 nmol/l; *p* = 0.072). LH values were higher in patients with FIB-4 upper the mean (7.0 IU/l vs 5.1 IU/l; *p* = 0.017) and a trend also observed when considering APRI score (7.1 IU/l vs 5.1 IU/l; *p* = 0.060).

**Table 2:** Reproductive hormones levels according to liver fibrosis scores in the cohort analyzed

	APRI			FIB-4		
	< 0.47 (# = 86)	≥ 0.47 (# = 21)	*p* value^a^	< 1.40 (# = 78)	≥ 1.40 (# = 29)	*p* value^a^
TT; median (IQR)	6.4 (5–8.2)	7.0 (5.5–9.4)	0.151	6.4 (4.9–8.6)	6.9 (5.3–8.3)	0.569
FT; median (IQR)	103 (69.6–126)	94.2 (68–117.8)	0.688	103 (71.7–129)	94.2 (66–116.3)	0.555
SHBG; median (IQR)	54 (38.7–69)	102 (53.1–118)	0.004	56 (38.5–70.5)	66 (44.1–106)	0.072
LH; median (IQR)	5.1 (3.8–8.1)	7.1 (3.9–11.9)	0.060	5.1 (3–7.9)	7 (4–13.2)	0.017

APRI and FIB-4 columns were divided according to the mean of APRI and FIB-4 in overall patients

*TT* total testosterone (ng/ml), *FT* free testosterone (pg/ml), *SHBG* sex hormone binding globulin (nmol/l), *LH* luteinizing hormone (U/l)

^a^*p* value calculation was done by Fisher exact test for categorial variables and Mann–Whitney test for continuous variables

### Correlation between TT, cFT, SHBG, LH and liver fibrosis

**Fig. 1:**
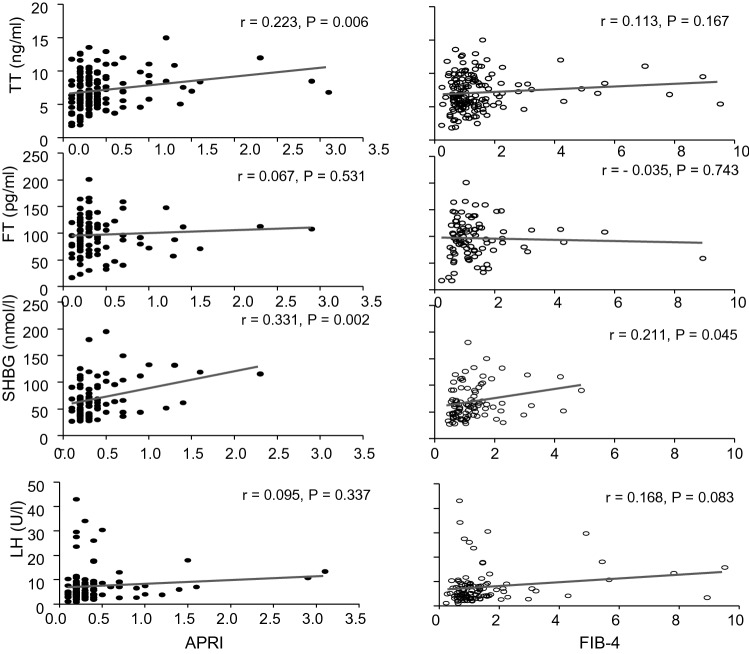
Correlation between TT, cFT, SHGB, LH and liver fibrosis

Finally, we analyzed the relationship between HPT markers and liver fibrosis. As showed in Fig. 1, a direct significant correlation was found between APRI score and TT (*r* = 0.223; *p* = 0.006) and SHBG (*r* = 0.331; *p* = 0.002); no correlation was found with cFT and LH levels. FIB-4 score positively correlated with SHBG (*r* = 0.211; *p* = 0.045). Multivariate analysis showed that only SHBG was independently associated with both liver fibrosis scores (APRI *p* < 0.0001; FIB-4 *p* = 0.002).

## Discussion

**Fig. 2:**
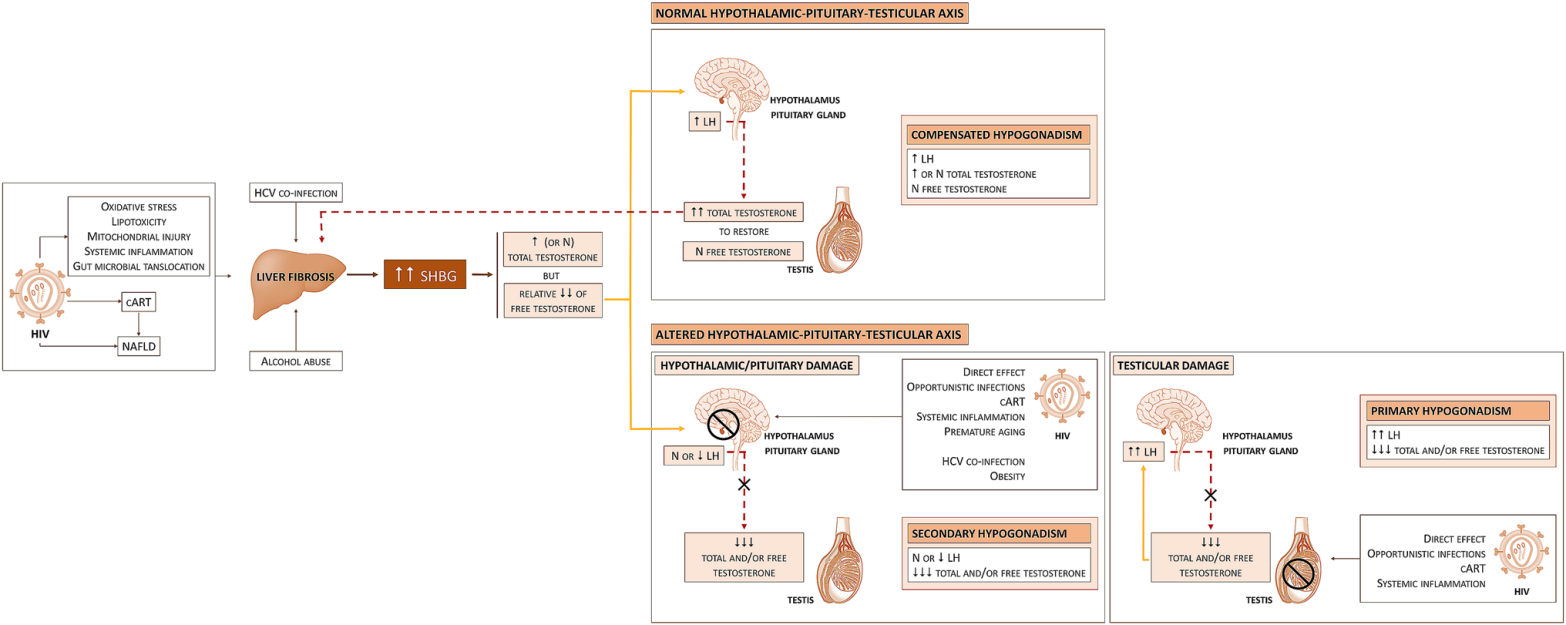
Potential impact of liver damage on the pathogenesis of hypogonadism in HIV-infected men. *HIV* human immunodeficiency virus, *cART* combined anti-retroviral therapy, *NAFLD* nonalcoholic fatty liver disease, *HCV* hepatitis C virus, *SHBG* sex hormone binding globulin, *LH* luteinizing hormone

Overall, in this cohort of HIV-infected patients the prevalence of hypogonadism was 30%. The most prevalent categories were compensated hypogonadism (15.8%) and hypogonadism with normal/low LH levels (11.2%). These two groups of patients showed characteristics suggesting a different pathophysiological mechanism. Compensated hypogonadism was associated with higher SHBG levels and liver fibrosis scores, together with longer duration of HIV infection. Furthermore, this group had also higher prevalence of detectable plasmatic HCVRNA. Importantly, liver fibrosis positively correlated with SHBG and TT levels as previously described [15–21]. As summarized in Fig. 2, our data suggest that in compensated forms T levels are maintained within normal range by an increase of LH production secondary to higher SHBG levels induced by liver fibrosis. SHBG level was associated to liver fibrosis independently of age, BMI, HCV ab or HIV duration. The maintenance of normal T concentrations relates to a normal HPT axis function, as pituitary gland should be able to respond to lower T levels induced by increased SHBG, and the testis should be able to increase steroidogenesis in response to LH. Therefore, the *primum movens* for hypogonadism onset in these patients seems to be liver dysfunction, as they show a normal function of HPT axis. The overall clinical and physiological significance of compensated hypogonadism is poorly understood. In a population‐based study of European men aged 40–79, compensated hypogonadism was associated with a highest frailty score [10]. In a big Italian study including 4173 patients consulting for sexual dysfunction, individuals with compensated hypogonadism reported psychiatric symptoms more often and had an increased predicted risk of cardiovascular events when compared with eugonadal individuals in general population [25].

On the contrary, in the second group of patients, with overt hypogonadism (low T levels) and normal/low LH values, LH is not increased and, therefore, the Leydig cell steroidogenic activity is not stimulated to maintain normal T production. In these patients, a certain grade of hypothalamic–pituitary dysfunction is plausible, so that there is an inadequate response to liver dysfunction and increased SHBG levels. Interestingly, patients in this group were more frequently obese, and it is well known that obesity is among the most frequent cause of secondary/normogonadotropic hypogonadism [26]. Focusing on the known body fat redistribution due to HIV-induced lipodystrophy, the relationship between body fat (not necessarily obesity) and gonadal status was recently evaluated by De Vincentis and colleagues [27]. They found that cFT and TT were inversely related, whereas oestradiol was directly related to total body and visceral fat, and therefore hypothesized that these findings could depend on an increase of testosterone aromatization, similarly to what happens in obese hypogonadal men of general population. Normogonadotropic hypogonadism is also called functional hypogonadism [22], indicating that low T levels occur in the absence of both intrinsic structural HPT axis pathology and of specific pathological conditions suppressing the HPT axis (e.g., prolactinoma). Indeed, HIV per se, obesity and increased SHBG are indicated as possible causes of functional hypogonadism [22, 28].

Systemic low chronic inflammation together with liver dysfunction, two frequent conditions in HIV population, might also be implied in the relative suppression of hypothalamus–pituitary function in this group of patients (Fig. 2).

Finally, primary hypogonadism was uncommon (2.8%) in our cohort of middle-aged HIV-infected patients, in agreement with previous reports [28]. Although the low number of patients affected by primary hypogonadism did not allow us detailed analyses, this form seems not to be related with liver fibrosis. In these cases, the primary testicular damage may be associated with a direct effect of HIV, opportunistic infections, long-term ART exposure and systemic inflammation on the testis [28] (Fig. 2).

Although many studies have been performed about hypogonadism in men with HIV, the cause and pathophysiological mechanisms of hypogonadism have not been clarified yet. Besides the usual risk factors for hypogonadism as the general population, HIV-related risk factors for hypogonadism include the duration of HIV infection, duration of ART, number of comorbidities, concomitant liver disease with HBV or HCV or non-alcoholic fatty liver disease, and presence of inflammatory cytokines such as tumor necrosis factor-α (TNF-α) or interleukin-1 [29–31].

Our study is the first to examine the possible involvement of liver dysfunction/fibrosis in hypogonadism among middle-aged HIV-infected patients, having also the advantage of considering LH levels as marker of hypothalamus–pituitary function and cFT as a more sensitive marker of hypogonadism than TT. Most of the studies on hypogonadism in patients with HIV-infection focused only on TT levels, very few included also gonadotropins, and none included liver fibrosis data [28].

The association between liver dysfunction and T metabolism is extensively described in the general population, but yet not fully understood [12, 14–21, 32]. Many factors may contribute to hypogonadism in cirrhosis, including hepatic overproduction of SHBG, changed SHBG isoforms with different steroid-binding affinities, elevated prolactin levels, direct suppression of Leydig cell function by estrogens or increased estrogen receptors in the liver [32]. A recent study [20] found that low cFT was associated with presence of NASH and severity of fibrosis in patients with histologically confirmed NAFLD. In agreement with this study, which was remarkably performed on liver biopsies, but also with another one based on non-invasive FIB-4 score [21], we showed that hypogonadism is frequent among middle-aged HIV-infected patients and it seems to be especially present in patients with high SHBG levels, liver fibrosis and HCV co-infection. These studies [20, 21], however, did not evaluate the HPT axis, therefore are not conclusive about the correlation between presence and/or grade of liver fibrosis and several classes of hypogonadism, especially because they did not study compensated hypogonadism forms.

Another strength of our study is the inclusion of only symptomatic patients. The diagnosis of hypogonadism should be based on the association between low T levels and clinical symptoms/signs of T deficiency, of which sexual symptoms are considered the most specific [22]. Indeed, most studies on HIV population did not consider hypogonadal symptoms, but rather assessed biochemical hypogonadism. In turn, this is important because the treatment of hypogonadism (usually with T replacement therapy, unless the patient is interested in fertility) is recommended only in symptomatic men with low T levels [22].

Interestingly, despite including only hypogonadal symptomatic patients, two thirds of them did not have a biochemical confirmation of any form of hypogonadism, suggesting that, at least in part, sexual symptoms can also be associated with other clinical and psychological HIV-infection-related aspects [33, 34].

We acknowledge some limitations in our study, including the small single-center cohort, the absence of determination of estradiol and albumin values, the lack of data on body composition and fat body distribution besides BMI and the absence of a control group without HIV-infection. Furthermore, this is a retrospective study and liver fibrosis assessment was performed with indirect scoring systems, such as the APRI and FIB-4 scores, instead of abdominal ultrasonography/elastography or liver biopsy, which is the gold standard both in general and HIV-infected populations [35]. However, also these methods have limitations: ultrasonography has limited accuracy in detecting fatty liver when < 35% of hepatocytes have been affected [36], and performing liver biopsy for patients who do not meet indications raises ethical considerations. Given the limitations of instrumental and histological methods for measurement of liver fibrosis, scores based on serum markers have been demonstrated good tools for the prediction of severity of liver fibrosis [37] and are widely used both in the general population and in HIV-infected patients [21, 36, 38–40].

## Conclusions

In conclusion, in a cohort of HIV-infected males complaining of sexual dysfunction, detailed endocrinologic evaluation detected hypogonadism in about one third of patients. Hypogonadism might be related to different pathophysiologic mechanisms, among which for the first time we found that liver fibrosis causing increase of SHBG was the *primum movens* of compensated forms of hypogonadism.

## Data Availability

Data are available upon motivate requests.
